# Hijacking a cellular highway: non-lipidated LC3 proteins and PCNT (pericentrin) drive influenza a virus uncoating

**DOI:** 10.1080/15548627.2025.2572527

**Published:** 2025-10-13

**Authors:** Yingying Cong, Fulvio Reggiori

**Affiliations:** a Department of Biomedicine, Aarhus University, Aarhus, Denmark; b Department of Drug Design, University of Groningen, Groningen, The Netherlands; c Department of Biomedical Sciences, University of Groningen, University Medical Center Groningen (UMCG), Groningen, The Netherlands

**Keywords:** Atg8ylation, centrosome, dynein, IAV cell entry, LC3-I

## Abstract

MAP1LC3/LC3 (microtubule associated protein 1 light chain 3) proteins have long been thought to carry out their cellular and organismal functions, including macroautophagy/autophagy, exclusively in their lipidated form, also referred to as Atg8ylation. They are anchored mainly to the phosphatidylethanolamine present in membranes through the action of two ubiquitin-like conjugation systems. Our recent work, however, uncovered a role of non-lipidated LC3s during influenza A virus (IAV) infection. We revealed that LC3s, together with the centrosomal scaffold protein PCNT (pericentrin), form a dynein adaptor complex that facilitates IAV uncoating at late endosomes (LEs). We also showed that co-opting the LC3s-PCNT complex is an alternative strategy to aggresome processing machinery (APM) hijacking via HDAC6, allowing IAV to exploit the force generated by dynein-dependent motors for virion uncoating and genome delivery in the host cytoplasm. Notably, the function of LC3s in IAV uncoating does not require their Atg8ylation or the core autophagy machinery, and PCNT’s role is independent from its centrosomal localization. These findings redefine LC3s as multifunctional adaptor proteins and reveal how viruses can co-opt centrosome assembly machinery components for host invasion.

**Abbreviation**: AKAP9/AKAP450- A-kinase anchoring protein 9; APM- aggresome processing machinery; IAV- influenza A virus; LC3s-I- non-lipidated LC3s; Les- late endosomes; MAP1LC3/LC3s-microtubule associated protein 1 light chain 3 proteins; MT-microtubule; NEU- neuraminidase; PCNT-pericentrin; TNPO1-transportin 1; vRNP-viral ribonucleoprotein

IAV is a membrane-enveloped virus with a segmented, single-stranded negative-sense RNA genome, which causes annual epidemics and occasional pandemics. Each of the eight genomic segments, together with multiple copies of viral nucleoprotein/NP and a single copy of the viral RNA polymerase, form the viral ribonucleoprotein (vRNP) complex. The membrane of IAV virions is studded with hemagglutinin/HA spikes, interspersed with NEU (neuraminidase) tetramers and a few M2 ion-channel proteins. Beneath this envelope, a layer composed of the matrix protein M1 shapes the particle and encases a core of vRNPs. IAV viral particles enter host cells via endocytosis and their fusion with the membrane of late endosomes (LEs) leads to uncoating, which is characterized by the separation of the viral membrane from vRNPs and concomitant release of the latter into the cytoplasm. Previous work has shown that the fusing IAV virions subvert the aggresome processing machinery (APM) by recruiting HDAC6 via unanchored ubiquitin chains on vRNPs, thereby activating actin- and microtubule (MT)-based forces to promote viral uncoating. However, HDAC6 depletion only partially inhibited IAV entry in the cytoplasm, suggesting the existence of redundant mechanisms. Our study has revealed that the adaptor complex formed by non-lipidated LC3s (LC3s-I) and PCNT physically links vRNPs to dynein, thereby driving IAV uncoating as well.

The conjugation of mammalian Atg8-family proteins (ATG8s), LC3A, LC3B, LC3C, GABARAP, GABARAPL1 and GABARAPL2, mostly to lipids but also to proteins is often referred to as Atg8ylation. However, evidence suggests a different functional scenario. In particular, a few reports indicate, for example, a role for LC3s-I in the endoplasmic reticulum-associated degradation/ERAD tuning pathway. However, the precise functions of the non-lipidated form of ATG8 proteins in this and other pathways remain poorly understood. In our study [1], we revealed that LC3s-I, but not non-lipidated GABARAP proteins, can function as part of a specialized, virus-engaged dynein adaptor complex that facilitates IAV cytoplasmic entry. This function does not depend on other key components of the autophagy machinery, such as ATG7, ATG13 and ATG16L1. One may thus hypothesize that the role of LC3s-I as part of one or more dynein adaptor complexes could be part of other cellular processes that require a functional association with MTs. For example, LC3s-I may mediate the trafficking of the ERAD tuning pathway vesicles with which they are associated. Additionally, those functions may be hijacked by pathogens. In fact, LC3s-I have been found to be associated with the membranous replication platforms of mouse hepatitis virus/MHV, equine arteritis virus/EAV and Japanese encephalitis virus/JEV. Similarly, inclusions generated by *Chlamydia trachomatis* are also decorated by LC3s-I. Therefore, a speculative idea is that these microbes co-opt LC3s-I and MT-mediated transport to guide the precise subcellular distribution of their components.

Lipidated mammalian ATG8 proteins interact with several dynein adaptors and mediate autophagosome trafficking. This observation underscores the inherent ability of ATG8 proteins to link intracellular cargoes to MT-based transport. We now show that LC3s-I possess this characteristic as well. We also observed that LC3s-I are recruited to LEs only during IAV cytoplasm entry, indicating that this recruitment is dictated by determinants within the IAV particles. In agreement with this notion, we detected an interaction between vRNPs and LC3s-I in immunoprecipitation experiments. It remains unknown which component(s) of the vRNPs are bound by LC3s-I

PCNT is a canonical component of the pericentriolar material/PCM, essential for centrosome organization, MT nucleation, and mitotic spindle assembly. It is also a dynein adaptor protein that transports specific centrosomal components along MTs to centrosomes. We observed that LC3s bind significantly less dynein upon PCNT depletion, and that vRNP proximity to PCNT is strongly reduced in the absence of LC3s, suggesting a binding axis of vRNP-LC3s-PCNT-dynein. PCNT contains a pericentrin-AKAP9/AKAP450 (A-kinase anchoring protein 9) centrosomal targeting/PACT motif, which is instrumental for its centrosomal localization, but this domain and therefore association with centrosomes is not important for IAV infection. Thus, our results have revealed an unexpected role of PCNT that can be co-opted by IAV for virion uncoating at LEs. Because PCNT functions as a ferry, carrying centrosomal materials along the MTs to centrosomes via interaction with dynein, vRNP-LC3s may also hijack this track to ensure the successful transport of vRNPs close to the nucleus, where they can interact with the nuclear pores and enter the nucleus where IAV replication starts.

While individual silencing of either HDAC6, PCNT or LC3s only partially impairs IAV cytoplasmic entry, dual knockdown of HDAC6 and PCNT or LC3s, but not just of PCNT with LC3s, phenocopies the complete inhibition seen with dynein depletion (Figure 1). We examined the levels of both the HDAC6 and LC3s-PCNT dynein adaptor in different cell types to determine whether their expression varies and might explain why IAV can enter the cell cytoplasm in two ways. Consistent with their roles in key housekeeping processes, we found that these proteins are ubiquitously expressed, suggesting that IAV may have adopted a dual strategy to increase the chances of infecting host cells. Importantly, we also discovered that this dual strategy is only conserved in the IAV strains belonging to the IAV phylogenic subgroup 1. In contrast, subgroup 2 strains exploit the LC3s-PCNT adaptor but not the HDAC6-APM system and employ a still-unknown dynein-independent mechanism. This observation underscores the need for caution when generalizing findings derived from a single IAV strain or a limited number of cell types.

**Figure 1.**
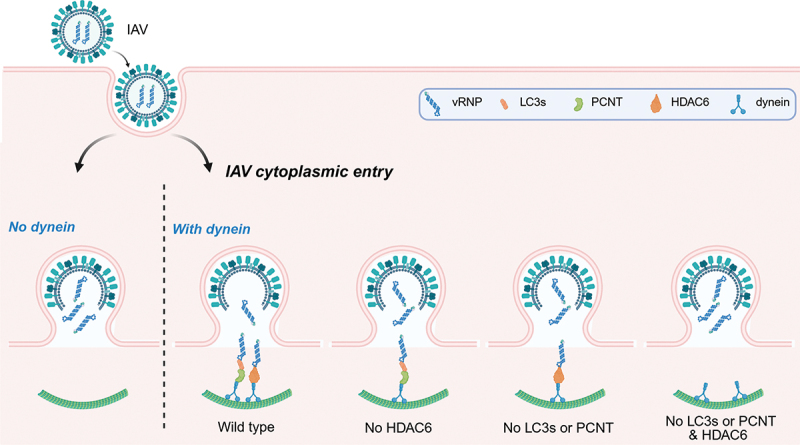
Model for IAV entry into the host cell cytoplasm. IAV virions enter host cells via endocytosis, and at LEs the virions fuse with the limiting membrane of this organelle, releasing the vRnps into the host cell cytoplasm. This IAV uncoating process is dynein-dependent and mediated by two redundant dynein adaptor systems, i.e., LC3s-PCNT and HDAC6. Individual loss of either HDAC6, LC3s or PCNT only partially impairs IAV cytoplasmic entry, whereas simultaneous disruption of both dynein adaptor systems phenocopies the effect of dynein depletion, leading to a complete block of vRNP cytoplasmic release. This figure is generated by BioRender with agreement no. JQ28GL5OJS.

In summary, IAV employs two parallel and redundant dynein-dependent viral uncoating pathways to ensure efficient entry in the host cytoplasm (Figure 1). In addition to the known host cell’s APM, we have described the LC3-PCNT complex as an adaptor that links vRNPs to the dynein motor for viral uncoating at LEs. This dual strategy highlights how IAV hijacks host motor and adaptor proteins to maximize the chances of a successful infection.

## Data Availability

Data sharing is not applicable to this article as no data were created or analyzed in this study.
